# Effects of Swallowing Cherry Stones

**Published:** 1811-06

**Authors:** Charles Batham

**Affiliations:** Cowick


### *To the Editors of the Medical and Physical Journal.*

Gentlemen,

YOUR Journal being a Miscellany for every principal as well as every collateral branch of medical science, likewise for every novelty in natural and philosophical history ; every case in medicine or surgery, which conveys information or novelty in practice, from whatever part of the world it may arise, does, and ought to find a place for the good of mankind, in your useful publication of so extended a range.

I should not have troubled you with the following case, but for a communication, in Journal 143, of Dr. Harrison from his son in America, on the fatal effects of eating cherries ; and the observations of Senex on the treatment, which whether right or wrong I shall not comment upon ; nor have I any doubt as to the truth of Dr. Harrison's relation of the case, or the practicability of opening the arteries at the wrists or ankles.

I will briefly state a case, and the method of treatment, which occurred to me last summer at the time of the cherry season. The mother of a young woman applied to me for her daughter, about twenty years of age, who had eaten voraciously of ripe cherries and swallowed the stones; soon after she complained of great uneasiness, pain, weight and nausea at her stomach, with tension and soreness of the abdomen, so that she was obliged to leave the place of her employment and return home, a few miles in the country. On the following day I was applied to, and ordered her an emetic of ipecac. and antim. tartariz. and a cathartic of pulv. jalap. and zingib.; the emetic to be taken immediately, and the purge a few hours after its operation, and repeated till copious evacuations by the bowels had been obtained; these had the desired effect, and in a few days she returned to her employment again perfectly well, after taking three doses of the purging physic. I enquired whether, during the operation of the emetic, she ejected from the stomach any cherry stones, but not one came up; nor did she know whether they passed per anum as the *cloaca* was inconvenient for observation; but that they did pass there can be no doubt, as such substances usually do pass the canal undigested. Whether the *rationale medendi* here pursued would have had the same good effect in Dr. Harrison's case, not being attended with convulsions, I am not sanguine enough to say, but leave it to your numerous class of pathological readers to form their own opinions upon.

Another case of repletion I shall relate from memory, (having mislayed my notes) which fell under my inspection in the summer, 1790, while on a tour in Ireland.

The history of the case was this; a boy, eight years of age, son of a labouring Irishman, having eaten great quantities of new wheat at the time of harvest which he had gleaned, until he was very ill, the parents brought him to me for advice. He was much swollen in the abdomen, face flushed, skin very hot, and painful to the touch, difficult respiration, and extremely restless, the state of his pulse I do not recollect; my attention was directed by the mother to a tumour in the posterior part of the perinæum, with the anus so much dilated that I could perceive the wheat therein, which surprised me much: I paused a little; but on recollecting what the late justly celebrated Dr. Hunter had said in his lectures on the abdominal viscera, in an impressive story respecting his saving the life of an old woman, labouring under a complaint of the ball stool, by manual operation, I instantly determined on attempting evacuation by mechanical means as far and as fast as I could; for this purpose I

took took the blunt end of a silver probe and bent it, so as to form a small obtuse angle, and sometimes the handle of a tea-spoon; with these I picked out the wheat as far as I could reach, which seemed to relieve the child much, and ordered the mother to bring the child again as soon as she found the tumour again formed; which happened in a few hours. I used the same method, and at the same time gave it a purge of pulv. jalap. & scammon. c calomel. By a few repetitions of these means the boy was soon restored, and I presume his life saved.

It is to be observed that the poor in Ireland see but little bread, therefore, it is not to be wondered at the child's eating so greedily and so much of such a rarity as wheat, and so impacted was it in the rectum, and swelled from the moisture of the bowels, that it was with great difficulty it could be moved.

If you think these cases worthy a place in your publication, by inserting them, you will oblige your constant reader,

CHARLES BATHAM, M.D.

*Cowick, April* 12, 1811.

